# Differential induction of malaria liver pathology in mice infected with *Plasmodium chabaudi* AS or *Plasmodium berghei* NK65

**DOI:** 10.1186/s12936-017-2159-3

**Published:** 2018-01-09

**Authors:** Diletta Scaccabarozzi, Katrien Deroost, Yolanda Corbett, Natacha Lays, Paola Corsetto, Fausta Omodeo Salè, Philippe E. Van den Steen, Donatella Taramelli

**Affiliations:** 1 Department of Pharmacological and Molecular Sciences, Università degli Studi di Milano, Milan, Italy; 2 Rega Institute for Medical Research, KU Leuven-University of Leuven, Louvain, Belgium; 3 Present Address: The Francis Crick Institute, London, UK; 4 Present Address: Dipartimento di Bioscienze, Università degli Studi di Milano, Milan, Italy

## Abstract

**Background:**

Cerebral malaria and severe anaemia are the most common deadly complications of malaria, and are often associated, both in paediatric and adult patients, with hepatopathy, whose pathogenesis is not well characterized, and sometimes also with acute respiratory distress syndrome (ARDS). Here, two species of murine malaria, the lethal *Plasmodium berghei* strain NK65 and self-healing *Plasmodium chabaudi* strain AS which differ in their ability to cause hepatopathy and/or ARDS were used to investigate the lipid alterations, oxidative damage and host immune response during the infection in relation to parasite load and accumulation of parasite products, such as haemozoin.

**Methods:**

Plasma and livers of C57BL/6J mice injected with *Pb*NK65 or *Pc*AS infected erythrocytes were collected at different times and tested for parasitaemia, content of haemozoin and expression of tumour necrosis factor (TNF). Hepatic enzymes, antioxidant defenses and lipids content and composition were also evaluated.

**Results:**

In the livers of *P. berghei* NK65 infected mice both parasites and haemozoin accumulated to a greater extent than in livers of *P. chabaudi* AS infected mice although in the latter hepatomegaly was more prominent. Hepatic enzymes and TNF were increased in both models. Moreover, in *P. berghei* NK65 infected mice, increased lipid peroxidation, accumulation of triglycerides, impairment of anti-oxidant enzymes and higher collagen deposition were detected. On the contrary, in *P. chabaudi* AS infected mice the antioxidant enzymes and the lipid content and composition were normal or even lower than uninfected controls.

**Conclusions:**

This study demonstrates that in C57BL/6J mice, depending on the parasite species, malaria-induced liver pathology results in different manifestations, which may contribute to the different outcomes. In *P. berghei* NK65 infected mice, which concomitantly develop lethal acute respiratory distress syndrome, the liver tissue is characterized by an excess oxidative stress response and reduced antioxidant defenses while in *P. chabaudi* AS infected mice hepatopathy does not lead to lipid alterations or reduction of antioxidant enzymes, but rather to inflammation and cytokine burst, as shown earlier, that may favour parasite killing and clearance of the infection. These results may help understanding the different clinical profiles described in human malaria hepatopathy.

**Electronic supplementary material:**

The online version of this article (10.1186/s12936-017-2159-3) contains supplementary material, which is available to authorized users.

## Background

Despite large investments and global interventions over the last century, malaria remains a leading cause of morbidity and mortality in humans with 212 million clinical cases in 2015 and 429,000 deaths estimated worldwide, 70% of which are children under 5 year of age [1]. The malaria infection can occur with fever and generalized sickness, and progress to severe anaemia or cerebral malaria, or can be asymptomatic. This range of clinical courses can be attributed to the host or to the parasites. In particular, the genetic background and the immune system of the host, the dynamics of transmission and the virulence of the parasites have to be considered [2, 3]. Hepatic involvement in cases of malarial infection is a well-recognized entity. Malaria hepatocellular dysfunction is characterized by an increase in the serum levels of bilirubin and aminotransferases, exceeding three times the upper limit of normal values. Malarial hepatopathy is associated with a higher incidence of cerebral malaria, shock, acute respiratory distress syndrome (ARDS) and acute kidney injury [4]. It occurs both in adult and paediatric patients, although it is more common in adults [3, 4]. One suspected cause of malarial hepatopathy is related to the activation of liver macrophages that phagocytize haemozoin or parasitized red blood cells [5]. Focal hepatocyte necrosis, cholestasis and granulomatous lesions have been documented in all malarial nodules [4]. In malaria patients with jaundice, histopathological changes in the form of damaged hepatocytes, congestion of liver cells, haemozoin deposition, inflammatory infiltrates, and cholestasis have been demonstrated [6, 7], as well as hyperplastic Kupffer cells [8].

The molecular basis of different manifestations of malaria using two species of murine *Plasmodium*, namely *Plasmodium berghei* strain NK65, and *Plasmodium chabaudi* strain AS have been recently investigated. *Plasmodium berghei* NK65-infection results in lethal ARDS [9], whereas *P. chabaudi* AS-infection in C57Bl/6J is self-healing after an initial peak of parasitaemia and recrudescence, and does not induce lung pathology [10, 11]. *Plasmodium berghei* NK65 and *P. chabaudi* AS parasites produce different amounts of haemozoin (Hz), and it has been demonstrated that Hz is pathogenic in the lungs by inducing pulmonary inflammation [12, 13]. Moreover, in mice infected by *P. berghei* NK65, the development of ARDS is associated with biochemical modifications and lipid alterations of lung tissue and disruption of the molecular organization and lipid composition of alveolar surfactant [12].

Excess Hz deposition was shown in the livers of *P. berghei* NK65-infected mice compared to *P. chabaudi* AS mice, while hepatomegaly due to inflammation and cell recruitment was higher in *P. chabaudi* AS mice. Just as in the lungs, liver dysfunction with elevated serum ALT and AST levels and production of both pro- and anti-inflammatory cytokines were associated with the amount of haemozoin present in the livers of *P. chabaudi* AS-infected mice. It was intriguing that inflammation, measured as cell recruitment and cytokine production, was much less pronounced in *P. berghei* NK65 livers that had higher amounts of haemozoin than *P. chabaudi* AS livers. Since haemozoin, due to its haem iron content, is strongly pro-oxidant and immunosuppressive [14, 15] the present work aimed at investigating the extent of oxidative damage, the lipid alterations and the antioxidant defences present in the liver tissue of C57BL/6J mice infected with *P. berghei* NK65, compared to mice infected with *P. chabaudi* AS.

## Methods

### Reagents

Unless otherwise specified, all biochemicals were purchased from Sigma (Milan, Italy). Standard fatty acid methylesters were purchased from Alltech (Milan, Italy), silica gel plates (Kieselgel 60) and high performance thin layer chromatography (HPTLC) from Merck (Darmstadt, Germany).

### Animals

The infection of C57BL/6J mice were performed by intraperitoneal injection of 10^4^ *P. berghei* NK65 or *P. chabaudi* AS infected red blood cells, as described [9, 12]. Three different experiments were performed; 6 or 8 mice/group (control and infected mice) were used for each time point and used at day 8 or 10 after infection. The time intervals and the dose of parasites were chosen based on previous experience. All the data were in line with and confirmed our previous study [16]. Mice were euthanized by intraperitoneal injection of a lethal dose of sodium pentobarbital (200 µl at 60 mg/ml Nembutal) at day 8 or 10 post infection and blood was removed by heart puncture and centrifuged to separate plasma and RBC.

### Preparation of biological specimens

Mice were perfused with 0.15 M NaCl containing 0.2 mM butylhydroxytoluene (BHT) as an antioxidant, and livers were removed, weighted and homogenized in Precellys tubes in six volumes of a solution containing 20 mM Tricine, 250 mM sucrose, 5 mM EDTA, 0.2 mM BHT (pH 7.4) and a protease inhibitor cocktail (Sigma, Milan, Italy).

### Haemozoin determination

The content of Hz in the perfused livers was analysed by haem-enhanced chemoluminescence determination according to Deroost et al. [13, 17]. The amount of Hz (pmol/mg tissue) was multiplied by the total weight of the liver and expressed as pmol or nmol haematin/organ.

### Lipid analyses

The extraction of lipids in the liver was performed according to Folch [18]. Dried lipid extracts were dissolved in chloroform and saved at − 80 °C until further analysis. The content of phospholipid (PL) phosphorus was determined by the Bartlett procedure [19]. Cholesterol (Cho), triglycerides (TG) and cholesterol esters (ChoE) were quantified by densitometric analysis (Camag Reprostar 3) after separation by HPTLC in hexane/diethyl ether/acetic acid (90:10:1 by vol.). A solution of 10% CuSO_4_ in 8% H_3_PO_4_ was used to visualize the spots. The fatty acid composition of the lipid fractions was analysed by gas liquid chromatography according to Corsetto et al. [20]. The levels of thiobarbituric acid reactive substances were used to determinate the lipid peroxidation, as previously reported [21] and expressed as pmoles of malondialdehyde (MDA).

### Antioxidant enzymes and other analyses

Antioxidant enzymes and total glutathione (GSH + GSSG) were determined in the supernatant of liver homogenates obtained after a centrifugation at 12000×*g* for 10 min. Protein concentration was determined by the Bradford assay (Bio-Rad, Hercules, CA) [22]. Catalase (CAT), glutathione reductase (GR) and superoxide dismutase (SOD) activity was determined according to Aebi et al. [23], Pinto et al. [24] and by a assay kit (Cayman Michigan, USA), respectively. Total GSH was determined spectrophotometrically by titration with 5-5′-dithiobis-2 nitro benzoic acid (DTNB) according to Beutler [25]. The collagen content of livers, was determined by measuring hydroxyproline in the tissue homogenates [26]. Liver damage was determined by measuring plasma levels of serum alanine transaminase (ALT) and aspartate transaminase (AST) enzymes, according to manufacturer’s protocol (Teco Diagnostics, California, USA).

### Quantitative reverse transcription-polymerase chain reaction

The liver homogenates were used to extract and quantify total RNA and to synthetize cDNA. A quantitative PCR was performed on 25 and 12.5 ng cDNA with primer and probe sets from Applied Biosystems according to Deroost et al. [13]. Data were normalized to 18S ribosomal RNA levels.

### Statistical analysis

The data are reported as mean ± standard deviation (SD). ANOVA test followed by Tukey’s multiple comparison test with GraphPad Prism 6.0 (Graphpad Software Inc., La Jolla, CA, USA) were performed to compare the groups and replicates. The differences were considered significant when p < 0.05. To eliminate possible artefacts (over- or under-estimation) due to hepatomegaly and hepatic oedema, all the biochemical parameters have been expressed as total content/liver.

## Results

### Malaria infection in mice

**Fig. 1:**
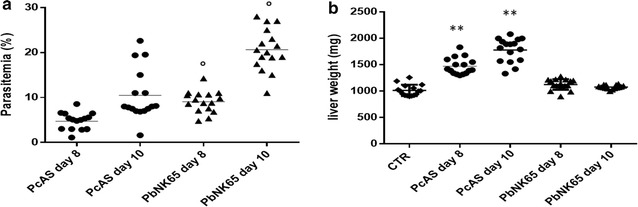
Disease course in mice infected with *P. berghei* NK65 or *P. chabaudi* AS. C57BL/6J mice were injected intraperitoneally with 10^4^ erythrocytes infected with *P. berghei* (*Pb*NK65) or *P. chabaudi* AS (*Pc*AS). Peripheral parasitaemia (**a**) and weights of the livers (**b**) were determined at day 8 and 10 post infection. n = 15–16 mice for each time point and strain. Data are pooled from three different experiments performed in the same conditions. **p < 0.01; vs control; °p < 0.05 *P. berghei* NK65 vs *P. chabaudi* AS

C57BL/6J mice were infected intraperitoneally with 10^4^ infected RBC (either *P. berghei* NK65 or *P. chabaudi* AS) and the development of parasitaemia was monitored every day whereas the liver pathology was checked on day 8 and 10 after the start of infection. These time intervals and the parasite dose were chosen on the basis of the indications from previous experiments [9]. Parasitaemia was lower in *P. chabaudi* AS compared to *P. berghei* NK65 infected mice at both time points (Fig. 1a), whereas hepatomegaly was evident in *P. chabaudi* AS, but not in *P. berghei* NK65-infected mice, corroborating previous data [16] (Fig. 1b). Similarly, elevated levels of AST and ALT were detected in the serum of infected mice at both time points (Additional file 1, Panel A and B). Of notice is the observation that in *P. berghei* NK65 infected mice, AST levels were particularly increased over ALT levels, leading to an AST/ALT ratio of 3.9 and 5.9 at day 8 and 10, respectively. Differently, in *P. chabaudi* AS infected mice the AST/ALT ratios was 1.5 and 1.2 at the same time intervals (Additional file 1, Panel C). These findings are consistent with the levels of parasitaemia in *P. berghei* NK65 mice since elevated AST levels may also reflect haemolysis in addition to liver damage [27]. Previous reports in a larger set of experiments confirm these observations. [16].

**Fig. 2:**
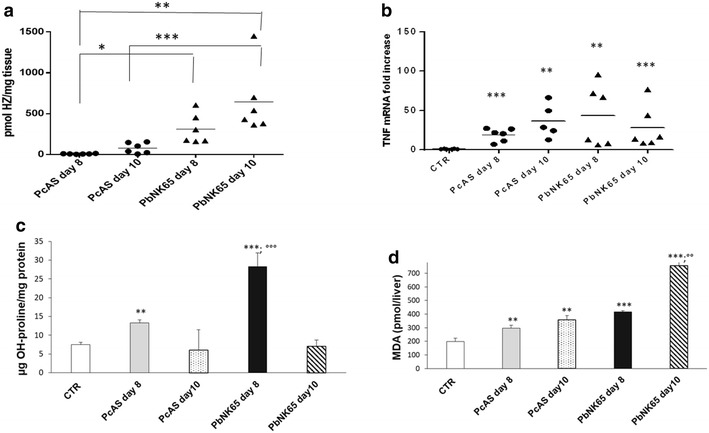
Hepatic haemozoin levels, oxidative damage and fibrosis in liver tissue of *P. berghei* NK65 or *P. chabaudi* AS infected mice. Hz content in liver tissue (pmol Hz/mg liver tissue) of *P. berghei* NK65 or *P. chabaudi* AS infected C57Bl/6J mice at day 8 and 10 post infection (**a**). Hepatic mRNA expression levels of TNF-α in non-infected and infected mice at day 8 or day 10 post infection determined by quantitative RT-PCR and normalized to 18S ribosomal RNA levels (**b**). OH-proline content (μg/mg protein) in non-infected and infected mice at day 8 or day 10 post infection (**c**); MDA content (pmol/liver) in non-infected and infected mice at day 8 or day 10 post infection (**d**). n = 6 mice/for each time point and group. *p < 0.05; **p < 0.01; ***p < 0.0001 vs Control; °°p < 0.05; °°°p < 0.01 *P. berghei* NK65 vs *P. chabaudi* AS

Differently from *P. chabaudi* AS-infected mice, livers from *P. berghei* NK65-infected mice also showed a dark-brown coloration in agreement with the significantly higher hepatic Hz deposition (Fig. 2a). In parallel with these modifications, also the proinflammatory status of the liver changed during the infections with the different *Plasmodium* species. The mRNA levels of TNF increased during the progression of the disease in the livers of both *P. berghei* NK65 and *P. chabaudi* AS infected mice (Fig. 2b), but with a different kinetic and values. Collagen deposition, measured as hydroxyproline content, was significantly higher with both parasite species at day 8 post infection with the highest levels in *P. berghei* NK65 livers. At day 10, the values returned to normal in both groups of mice, as those of control (Fig. 2c). Lipid peroxidation, expressed as total pmoles MDA/liver, was found to be significantly and time-dependently increased in mice infected with both *Plasmodium* species showing higher levels in *P. berghei* NK65 infected mice at either day post infection (Fig. 2d).

### Oxidative stress response in the liver

**Fig. 3:**
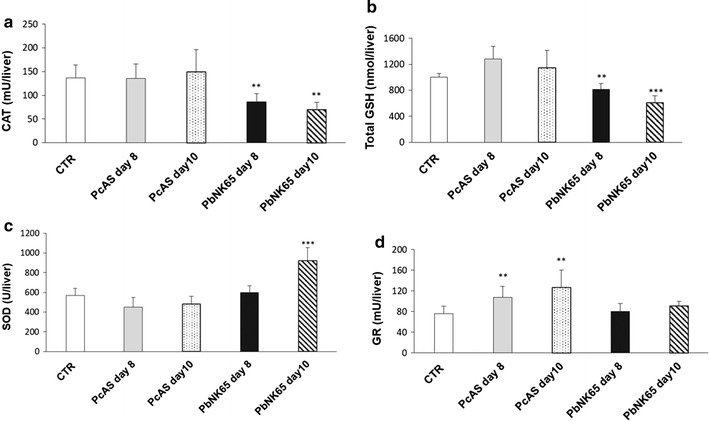
Enzymes activity in the liver of C57Bl/6J mice infected by *P. berghei* NK65 or *P. chabaudi* AS. Catalase activity (**a**), total GSH (**b**), SOD activity (**c**) and glutathione reductase activity (**d**) were determined in the livers of non-infected and infected mice at day 8 and day 10 post infection. n = 8 mice/group. Data are from one representative experiment out of three performed in the same conditions; *p < 0.05; **p < 0.01; ***p < 0.0001 vs control

The above findings, namely the increased levels of Hz and increased levels of MDA suggestive of oxidative stress in *P. berghei* NK65-livers, prompted us to investigate the status of the antioxidant defence systems in the infected livers as a determinant of tissue susceptibility to oxidative damage. The pattern of response in the two groups of infected mice was again different. As expected from the higher levels of lipoperoxidation, the liver extracts of *P. berghei* NK65 infected mice were characterized by a lower CAT activity and lower levels of total glutathione (GSH + GSSG) at both days post infection (Fig. 3a, b). SOD activity was significantly enhanced at day 10 post infection, whereas GR was not different from uninfected mice (Fig. 3c, d). On the contrary, *P. chabaudi* AS-infected mice showed CAT and SOD activity and GSH levels similar to control at both days post infection, whereas GR activity was higher at both time points (Fig. 3d).

### Species-specific lipid changes in infected livers

**Fig. 4:**
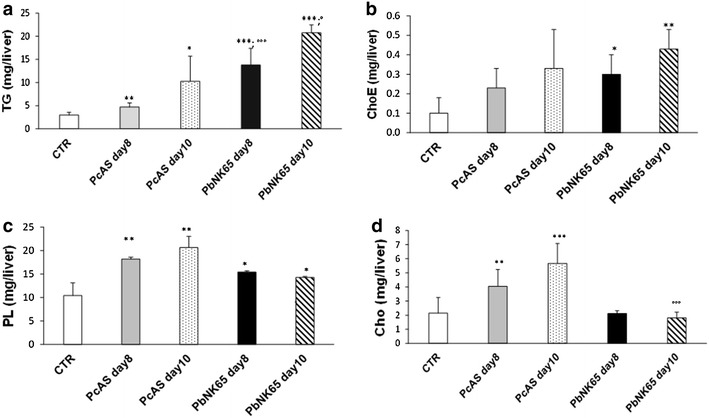
Alteration in the lipid profiles of the livers from uninfected, *P. berghei* NK65 or *P. chabaudi* AS infected mice. Mice infected with *P. berghei* NK65 or *P. chabaudi* AS were dissected at day 8 and 10 post infection. The total content of liver triglycerides (TG) and phospholipids (PL) were determined as described in “Methods” (**a** and **c**). The content of ChoE and free Cho is shown in **b** and **d**, respectively. n = 8 mice for each condition. Data are from one representative experiment out of three performed in the same conditions *p < 0.05; **p < 0.01; ***p < 0.0001 vs Control; °p < 0.05; °°°p < 0.001 *P. berghei* NK65 vs *P. chabaudi* AS

**Fig. 5:**
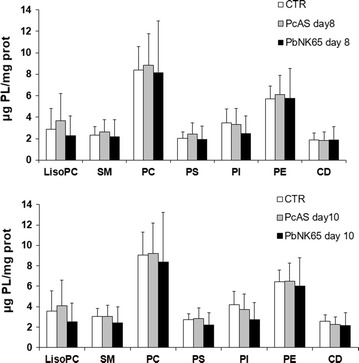
Phospholipids composition in liver from uninfected, *P. berghei* NK65 or *P. chabaudi* AS infected mice. The distribution of liver PL (µg PL/mg protein) was determined in uninfected and in *P. berghei* NK65- or *P. chabaudi* AS-infected mice at day 8 or 10 post infection. Data are from one representative experiment out of three performed in the same conditions. *LPC* lysophosphatidylcholine, *SM* sphingomyelin, *PC* phosphatidylcholine, *PS* phosphatidylserine, *PI* phosphatidylinositol, *PE* phosphatidylethanolamine, *CD* cardiolipine

Liver dysfunction is generally associated with lipid alterations. The lipid profile of infected livers was significantly modified compared to control mice and different between the two species. TG levels were significantly higher in the *P. berghei* NK65- compared to *P. chabaudi* AS-infected livers (Fig. 4a). In addition, higher levels of ChoE, and lower levels of free Cho were seen with *Pb*NK65 at both day 8 and 10 post infection, compared to control mice, whereas *P. chabaudi* AS-infected mice had higher levels of free cholesterol (Fig. 4b, c). Compared to control mice, PL were more strongly increased in *P. chabaudi* AS-infected livers than in *P. berghei* NK65 at both days post infection (Fig. 4d), whereas no differences were found in the composition of the PLs due to infection (Fig. 5).

Modification in the fatty acid distribution between *P. berghei* NK65 vs *P. chabaudi* AS livers was also observed. Palmitic acid was significantly increased in *P. chabaudi* AS livers compared to control, whereas this was decreased in *P. berghei* NK65 together with palmitoleic acid. Among the polyunsaturated fatty acids, significantly higher amounts of linoleic acid (C18:2 n − 6) and lower levels of eicosatrienoic (C20:3 n − 6) and arachidonic (C20:4 n − 6) acids were seen in *P. berghei* NK65 compared to control (Table 1). These differences were less pronounced in *P. chabaudi* AS livers and limited to the changes of arachidonic acid (C20:4 n − 6) and docosapentaenoic acid (C22:5 n − 3). As a consequence of these changes, also the C18:2/C20:4 ratio was significantly increased in *P. berghei* NK65 compared to control or *P. chabaudi* AS.

**Table 1:** Fatty acid distribution in the liver tissue of *Plasmodium chabaudi* AS or *Plasmodium berghei* NK65 infected mice at different days after the infection

	% of total				
	Control	*P. chabaudi* AS day 8	*P. chabaudi* AS day 10	*P. berghei* NK65 day 8	*P. berghei* NK65 day 10
C16:0 palmitic acid	20.76 ± 1.0	25.04 ± 0.8*	26.10 ± 0.1*	18.41 ± 0.9*	18.01 ± 0.2*
C16:1 palmitoleic acid	1.10 ± 0.2	1.24 ± 0.1	1.17 ± 0.1	0.63 ± 0.3*	0.59 ± 0.1*
C18:0 stearic acid	13.25 ± 1.6	12.80 ± 0.7	13.47 ± 1.1	13.54 ± 1.7	13.87 ± 0.9
C18:1 oleic acid	10.12 ± 1.5	9.98 ± 1.5	11.01 ± 1.2	10.52 ± 1.5	10.20 ± 1.0
C18:2 n − 6 linoleic acid	15.00 ± 1.4	17.4 ± 0.6	17.98 ± 1.2	22.19 ± 2.4**	23.01 ± 1.9**
C18:3 n − 3 α-linolenic acid	0.25 ± 0.1	0.22 ± 0.1	0.19 ± 0.1	0.21 ± 0.1	0.20 ± 0.1
C18:3 n − 6 γ-linolenic acid	0.17 ± 0.1	0.18 ± 0.1	0.17 ± 0.1	0.16 ± 0.1	0.19 ± 0.1
C20:3 n − 6 eicosatrienoic acid	1.20 ± 0.1	0.98 ± 0.2	1.10 ± 0.1	0.51 ± 0.1***	0.49 ± 0.1***
C20:4 n − 6 arachidonic acid	22.02 ± 1.1	19.99 ± 0.8*	18.97 ± 1.0*	16.81 ± 1.4***	16.01 ± 1.2***
C20:5 n − 3 eicosapentaenoic acid	0.29 ± 0.1	0.22 ± 0.1	0.30 ± 0.1	0.21 ± 0.1	0.29 ± 0.1
C22:5 n − 3 docosapentaenoic acid	0.38 ± 0.1	0.57 ± 0.1*	0.60 ± 0.2*	0.37 ± 0.1	0.39 ± 0.1
C22:6 n − 3 docosahexanoic acid	9.28 ± 0.7	9.26 ± 1.2	9.40 ± 1.0	9.79 ± 0.6	9.70 ± 0.9
C18:2/C20:4	0.68 ± 0.1	0.87 ± 0.1	0.95 ± 0.1*	1.32 ± 0.1**	1.44 ± 0.2***

Data are expressed as mean ± SD

* p < 0.05; ** p < 0.01; *** p < 0.001 vs Control n = 6–7

## Discussion

The pathogenesis of hepatic dysfunction in malaria is complex and not completely understood. Histopathological findings show congested and swollen hepatocytes, Kupffer cell hyperplasia, deposition of brown malarial pigment, mononuclear cell infiltration, micro-occlusion by parasitized RBC and, less frequently, steatosis and spotty and submassive necrosis [2, 7, 13, 16, 28]. Infection, inflammation and oxidative stress have been shown to induce multiple alterations in hepatic lipid and lipoprotein metabolism [29]. The present study demonstrates that blood stage malaria infection by *P. chabaudi* AS or *P. berghei* NK65 exerts different effects on the liver of C57BL/6J mice. Hepatomegaly was confirmed in *P. chabaudi* AS mice, as previously shown [16] as well as liver dysfunction suggested by the serum increase of the hepatic enzymes AST and ALT, especially at day 10 post infection. The high AST levels lead to an AST/ALT ratio significantly higher in *P. berghei* NK65 compared to *P. chabaudi* AS. The AST/ALT index is used to discriminate the type of liver damage observed [27, 30] and a progressive increase in the AST/ALT ratio correlated with a decrease in liver functions, like in cirrhosis and fibrosis [31]. Since plasma clearance of AST is modulated by the activity of sinusoidal liver cells, during progressive fibrosis and cirrhosis, the functions of these cells are progressively impaired resulting in a relative increase in AST levels [32]. In the case of malaria, in *P. berghei* NK65-infected mice, an alternative hypothesis can be proposed. Elevated AST levels in absence of elevated ALT, in fact, may indicate haemolysis, since RBC contain AST. This has already been reported in sickle cell disease where the AST/ALT ratio has been used as haemolytic index [33]. In the models used for this work, the AST/ALT ratio may well indicate initial fibrosis in both species, as confirmed by the OH-proline increase at day 8 post infection, and increased haemolysis in *P. berghei* NK65-infected mice that develop higher parasitaemia than *P. chabaudi* AS mice. The latter leads to higher levels of Hz in the liver of *P. berghei* NK65- than in *P. chabaudi* AS-infected mice [16]. It is well known that Hz plays a crucial role in generating oxidative damage [12, 14, 15]. The higher content of Hz, free (pro-oxidant) haem derived from intravascular haemolysis and high parasitaemia are likely to contribute to excessive oxidative stress response in *P. berghei* NK65 livers. TNF may also participate. A marginal role seems to be played by the inflammatory infiltrate, which is much less abundant in *P. berghei* NK65-infected livers than in *P. chabaudi* AS mice, in which increased liver weight, histology and the correlation between hepatic inflammation, enzymes and haemozoin were demonstrated [16].

The higher production of ROS in *P. berghei* NK65-infected mice was confirmed by the elevated concentration of MDA, which is an index for the loss of structure and cell membrane integrity, and the concomitant depletion of the antioxidant defence system, such as CAT and total GSH. GSH constitutes the first line of defence against free radicals and is a critical determinant of tissue susceptibility to oxidative damage. The increase of SOD, shown also in other models of liver damage [34], reveals the necessity of minimizing oxidative stress, which largely derives from the production of superoxide anion radicals. These changes seems to be associated with *P. berghei* NK65 infection, since they were not present in *P. chabaudi* AS-infected mice that showed only an increase of GR at both days post infection.

An important change observed in *P. berghei* NK65 vs *P. chabaudi* AS mice was the augmentation of the liver content of TG and cholesterol esters that indicates a potent stimulation of hepatic lipogenesis by *P. berghei* parasites [35]. This is in agreement with literature data showing that livers from *P. berghei*-infected mice contain lipid droplets and myelin-like figures [36]. A significant, although limited increase of TG was also present in *P. chabaudi* AS infected mice in agreement to the small neutral lipid inclusions observed by Seixas et al. [37]. Elevated TG are a characteristic of liver pathologies of different aetiology, clinically defined as non-alcoholic fatty liver disease in which TG accumulate and plasma albumin and protein decrease.

On the contrary, higher amounts of PL and free Cho were present in *P. chabaudi* AS-infected mice compared to contr or *P. berghei* NK65-infected mice. This is possibly related to reticuloendothelial hyperplasia and/or cholestasis, similar to those reported in the same or other models of malaria infection [7, 38]. The fatty acid distribution in the liver tissue of *P. chabaudi* AS or *P. berghei* NK65 infected mice at different days after the infection confirms the impairment (possibly induced by ROS) of the elongation/desaturation pathway from linoleic to arachidonic acid and may explain the higher linoleic/arachidonic acid ratio in the liver of *P. berghei* NK65-infected mice [39]. These observations also contribute to explain the changes in the lipid content and composition that was reported in the lungs of *P. berghei* NK65 infected mice [12].

All together, these findings are in agreement with other models of *P. berghei* infection and support the hypothesis that ROS may play a critical role in the liver dysfunction caused by *P. berghei* NK65 parasites [35, 40]. Consistent with this hypothesis are also the signs of fibrosis, revealed by the elevated hepatic levels of hydroxyproline at day 8 post infection in *P. berghei* NK65-infected mice. Indeed, it is well known that oxidative stress is one of the major stimuli of fibrosis [41]. Excess of ROS may induce severe liver damage followed by a phase of repair [42] during which TGF-β1 on one hand limits the proliferative response of hepatocytes, and on the other hand, increases the production of collagen and other extracellular matrix proteins. Submicroscopical signs of fibrosis were evident also in *P. chabaudi* AS-infected mice, although at a lower extent. Interestingly, at day 10 post infection, the amount of hydroxyproline decreased with both parasite species, becoming similar to control mice. The hypothesis that inflammation may lead to the activation of matrix metalloproteinases (MMPs) is under investigation. MMPs are proteolytic enzymes able to degrade different proteins of the extracellular matrix (e.g. collagen, laminin, fibronectin) and modulate cytokine and chemokine activity in cases of severe inflammation. Previous data indicated that, in the liver of C57Bl/6J mice infected with *P. berghei* ANKA, the activity of MMPs is significantly increased [43]. Furthermore, several in vitro studies have shown a TNF-dependent induction of MMPs at both mRNA and protein levels in monocytes fed with natural Hz [16, 44].

## Conclusion

In conclusion, this study demonstrates that, in C57BL/6J mice, *P. berghei* NK65 and *P. chabaudi* AS parasite species induce liver pathology in a different manner. In particular, in *P. berghei* NK65 infected mice, which succumb of malaria ARDS, it seems that an excess oxidative response predominates, as demonstrated by the alteration of the liver anti-oxidant enzymes, the increased MDA levels and the accumulation of TG. These changes are related to the high parasitaemia, the liver Hz deposition and the modification in protein content and lipid profile. These alterations are less pronounced with *P. chabaudi* AS, which develops a self-limiting infection, with lower parasitaemia and Hz deposition, and thus less oxidative stress. The cell infiltrate and the cytokine burst occurred at the peak of the infection may suggest an inflammatory liver response, which precedes parasite clearance [16].

## Additional file

**Additional file 1.** ALT and AST determination in mice infected with *P. berghei* NK65 or *P. chabaudi* AS. C57BL/6J mice were injected intraperitoneally with 10^4^ erythrocytes infected with *P. berghei* NK65 or *P. chabaudi* AS. Serum levels of AST (panel A), ALT (panel B) and the AST/ALT ratio (panel C) were determined at day 8 and 10 post infection according to manufacturer’s protocol (Teco Diagnostics, California, USA). n = 3-6 mice for each time point and strain, additional data can be found in [16]. *p < 0.05; **p < 0.01 *versus* control.

## Abbreviations

ALT: alanine transaminase
ARDS: acute respiratory distress syndrome
AST: aspartate transaminase
BHT: butylhydroxytoluene
CAT: catalase
Cho: cholesterol
ChoE: cholesterol esters
GR: glutathione reductase
GSH + GSSG: total glutathione
HPTLC: high performance thin layer chromatography
Hz: haemozoin
MDA: malondialdehyde
*Pb*NK65: *Plasmodium berghei* NK65
*Pc*AS: *Plasmodium chabaudi* AS
PL: phospholipid
SOD: superoxide dismutase
TG: triglycerides
TNF: tumor necrosis factor
