# Supplementary figures and images for: Differential induction of malaria liver pathology in mice infected with Plasmodium chabaudi AS or Plasmodium berghei NK65
Source: Malar J. 2018 Jan 9;17:18. doi: 10.1186/s12936-017-2159-3 (PMC5761140; doi:10.1186/s12936-017-2159-3)

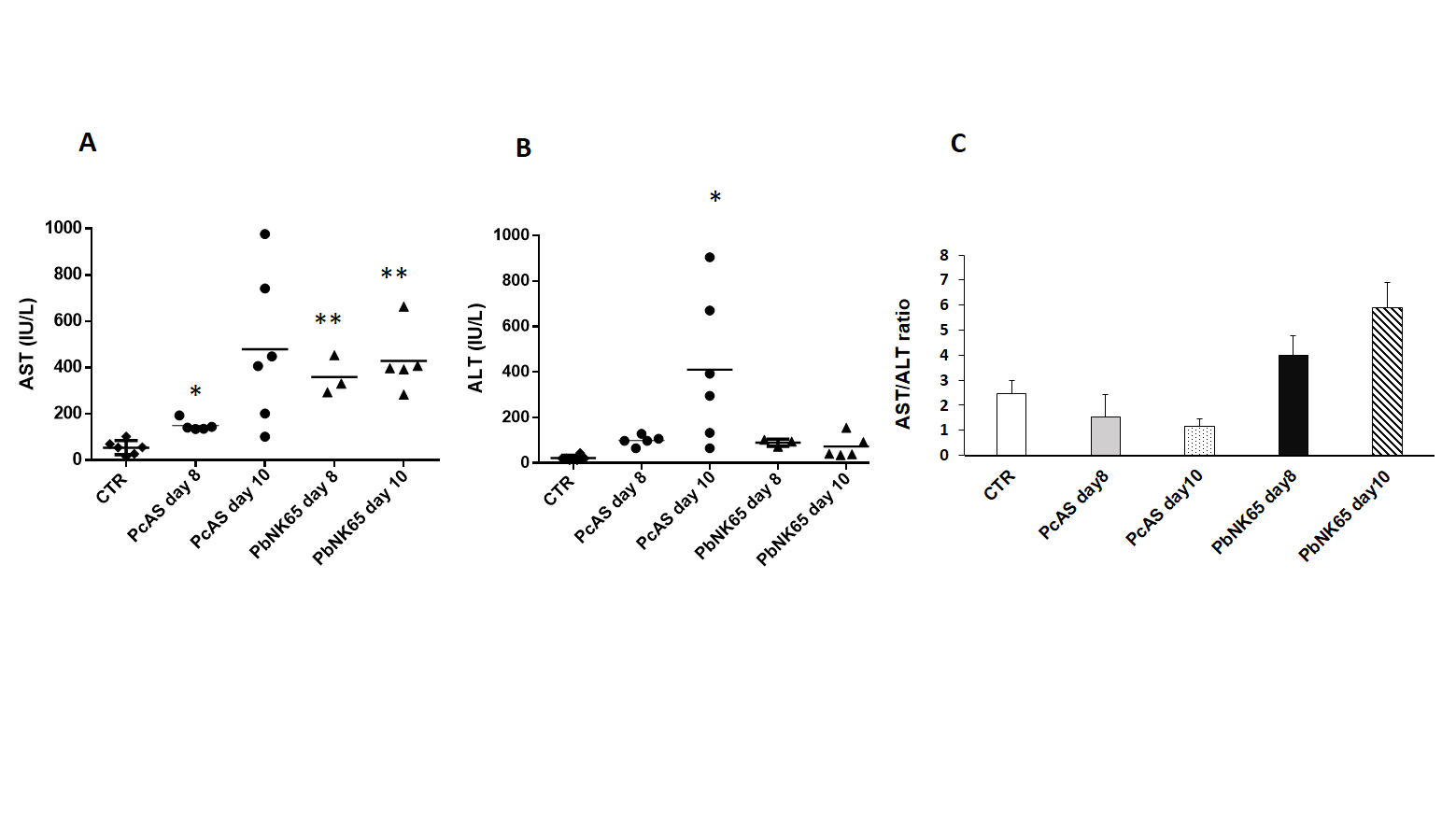

Supplement: Supplementary file 1 — Additional file 1. ALT and AST determination in mice infected with P. berghei NK65 or P. chabaudi AS. C57BL/6J mice were injected intraperitoneally with 104 erythrocytes infected with P. berghei NK65 or P. chabaudi AS. Serum levels of AST (panel A), ALT (panel B) and the AST/ALT ratio (panel C) were determined at day 8 and 10 post infection according to manufacturer’s protocol (Teco Diagnostics, California, USA). n = 3-6 mice for each time point and strain, additional data can be found in [16]. *p < 0.05; **p < 0.01 versus control. [file 12936_2017_2159_MOESM1_ESM.tif]
